# Comparison of visual quality between the refractive-diffractive hybrid bifocal EDOF and the refractive-diffractive hybrid trifocal IOL

**DOI:** 10.3389/fmed.2026.1872773

**Published:** 2026-07-29

**Authors:** Yanfeng Zeng, Qiyao Ma, Yuancheng Qu, Lie Ma, Jipu Bu, Xiaoli Zhou, Li Sheng, Shiping Yin

**Affiliations:** 1 Cataract Department, Lixiang Eye Hospital of Soochow University, Suzhou, China; 2 School of Medicine, Shanghai University of Medicine and Health Sciences, Shanghai, China; 3 Cataract Department, ChangSha BoShi Eye Hospital, ChangSha, China

**Keywords:** cataract, aspheric diffractive multifocal intraocular lens, vision, extended depth of focus refractive, patient satisfaction

## Abstract

**Purpose:**

To comparatively analyze visual quality after cataract surgery with implantation of the extended depth of focus (EDOF) refractive-diffractive hybrid bifocal intraocular lens (IOL) AM3UH and the refractive-diffractive hybrid trifocal intraocular lens AT LISA tri 839MP.

**Methods:**

This was a multicenter, retrospective and prospective bidirectional cohort study. Sixty-nine patients (69 eyes) with AM3UH IOL implantation were enrolled in the AM3UH group. Sixty-two patients (62 eyes) implanted with AT LISA tri 839MP IOL were enrolled in the 839MP group. Uncorrected distance visual acuity (UDVA, logMAR), uncorrected intermediate visual acuity (UIVA, logMAR), uncorrected near visual acuity (UNVA, logMAR) and best corrected distance visual acuity (BCDVA) were assessed before and 3 months after surgery. The visual function index 11R (VF-11R) was used to evaluate the quality of life after cataract surgery. Light interference, spectacle independence and postoperative satisfaction were evaluated by questionnaires.

**Results:**

The preoperative UDVA of AM3UH group and 839MP group was (0.93 ± 0.38) logMAR and (0.90 ± 0.47) logMAR, respectively. At 3 months postoperatively, the UDVA, UIVA, UNVA and BCDVA of the AM3UH group were (0.14 ± 0.19) logMAR, (0.13 ± 0.12) logMAR, (0.15 ± 0.1) logMAR, and (0.09 ± 0.11) logMAR, respectively; and those of the 839MP group were (0.90 ± 0.47) logMAR, (0.12 ± 0.08) logMAR, (0.07 ± 0.08) logMAR, (0.12 ± 0.06) logMAR, and (0.05 ± 0.04) logMAR, respectively. Both IOLs (AM3UH and 839MP) significantly improved postoperative UDVA. No significant differences were observed between groups regarding distance, near, or best-corrected distance visual acuity (*P* > 0.05); however, the trifocal 839MP group demonstrated significantly superior UIVA. Patient satisfaction with intermediate and near vision was also higher in the 839MP group, though far vision satisfaction was similar. Both lenses showed low and comparable levels of glare and halos (the majority of patients were either asymptomatic or reported only mild symptoms), with a slight trend toward less glare and more halos in the 839MP group. Rates of spectacle independence at all distances did not differ significantly (*P* > 0.05).

**Conclusion:**

Both lenses offer good visual quality and increased spectacle independence. The AT LISA tri 839MP provides superior intermediate vision and related patient satisfaction, with a similar overall side-effect profile.

## Introduction

With the rapid development of cataract surgery technology, the function of intraocular lens (IOL) is becoming increasingly more advanced. Cataract surgery with a refractive aim has gradually become the first-choice procedure for patients with cataract. Increasingly customized IOLs customized IOLs have been used in clinical practice. The AM3UH is an extended depth of focus (EDOF) refractive-diffractive hybrid multifocal intraocular lens (MIOL) manufactured from hydrophobic acrylic materials. The anterior optical surface adopts a higher-order aspheric design, with the central 1 mm zone being a refractive area corresponding to the paraxial region. This zone generates depth of field without perceptible spherical aberrations. Images remain clear on the retina, while achieving an extended depth of field. A large spherical aberration within the central 1 mm zone creates an EDOF, simulating the eye’s continuous accommodative function and providing continuous vision over a certain range of distances (1), thereby achieving an extended depth-of-field effect. Beyond the central 1 mm zone, the design gradually transitions to a normal spherical aberration profile. The smooth transition between these two zones helps reduce disturbances such as glare. The posterior optical surface features a diffractive bifocal design within a 4.0 mm diameter, with an additional dioptric power of +2.8 D to provide bifocal capability. The combination of the anterior and posterior surfaces aims to deliver clear and continuous vision, continuous vision across the full range of distances—far, intermediate, and near—thereby reducing the patient’s postoperative dependence on spectacles. The peripheral square edge design, frosted finishing, the third-generation aspheric design, and a reduced additional power of +2.8 D (compared to the standard +3.2 D) collectively enhance AM3UH’s resistance to tilt and decentration, minimizing glare and halos. The AT LISA tri 839MP is a refractive-diffractive hybrid trifocal IOL designed with a smooth aspheric profile. It is manufactured from hydrophilic acrylic materials and features a hydrophobic surface treatment. The optical zone diameter is 6.0 mm, with a central refractive zone of 1.04 mm in diameter surrounded by a diffractive region. The anterior optical surface incorporates a trifocal zone within the central 0–4.3 mm diameter and a bifocal zone in the peripheral 4.3–6.0 mm diameter area. It provides an additional degree of +1.66 D for intermediate vision and +3.33 D for near vision, supporting functional visual acuity at reading distance (40 cm) and intermediate distance (80 cm). Incoming light is distributed among far, intermediate, and near focal points in a ratio of approximately 50%, 20%, and 30%, respectively, ensuring adequate focus across all distances while enhancing intermediate vision (2). The incorporated light distribution technology is designed to minimize the occurrence of halos (3). To address the limitations associated with IOLs based on different optical principles, we selected two representative IOLs that combine distinct optical designs for comparison: the extended depth-of-focus refractive-diffractive hybrid bifocal MIOL AM3UH and the refractive-diffractive hybrid trifocal IOL AT LISA tri 839MP. This study aimed to evaluate postoperative visual quality following their implantation, thereby providing further evidence to guide the selection of IOLs for cataract patients in clinical practice.

## Materials and methods

### Study design and participants

A retrospective-prospective bidirectional cohort study was conducted. In this study, eligible patients were first enrolled based on predefined inclusion criteria. For these enrolled patients, we retrospectively collected their previously documented follow-up data (e.g., preoperative and early postoperative visits) from medical records. Subsequently, we conducted prospective follow-up from the time of enrollment onward. This study was conducted at two clinical centers. Patients who underwent cataract surgery and received the EDOF refractive-diffractive hybrid bifocal MIOL AM3UH at Lixiang Eye Hospital of Soochow University and ChangSha BoShi Eye Hospital between January 2024 and August 2024 were enrolled as the AM3UH group. The numbers of patients (eyes) enrolled from the two centers were 42 (42 eyes) and 27 (27 eyes), respectively, totaling 69 patients (69 eyes). Patients who received the Zeiss refractive-diffractive hybrid trifocal IOL AT LISA tri 839MP were enrolled as the 839MP group, with 40 (40 eyes) and 22 (22 eyes) from the two centers, respectively, totaling 62 patients (62 eyes). This study strictly adhered to ethical principles and was approved by the Medical Ethics Committee of the Lixiang Eye Hospital of Soochow University (SLER2024307). The study was conducted in accordance with the tenets of the Declaration of Helsinki. Written informed consent was obtained from all the included patients.

Inclusion criteria were: age ≥ 18 years; cataract with planned unilateral or bilateral IOL implantation; clear ocular media apart from the cataract and possible mild vitreous opacities; spherical equivalent of the implanted IOL between +6.0 D and +30.0 D.

Exclusion Criteria were: Preoperative corneal astigmatism >1.0 D or irregular corneal astigmatism.; retinal or macular diseases likely to affect visual acuity (including myopic macular degeneration and other retinal pathologies), glaucoma, traumatic cataract, congenital bilateral cataracts, and other severe intraocular conditions; corneal endothelial cell density below 2000 cells/mm^2^; inability to complete follow-up examinations.

### Surgical procedures

All procedures were performed by a chief physician with extensive clinical experience. In all cases, 0.3% levofloxacin eye drops were instilled preoperatively. After achieving full mydriasis using compound tropicamide eye drops, surgery was conducted under topical anesthesia with proparacaine hydrochloride eye drops. A 2.2 mm clear corneal primary incision was made, and a viscoelastic agent was driven through the incision. Next, capsulorhexis (5.0–5.5 mm in size) was performed on the anterior membrane of the lens with forceps, followed by a corneal-side incision, and hydrodissection to separate the lens cortex from the nucleus. Phacoemulsification was performed to extract the lens nucleus and cortex. The anterior and posterior capsules were thoroughly polished, and the IOL was implanted into the capsular bag through the main limbal incision. The viscoelastic agent was removed, and the corneal incision was sealed by hydration. The IOL position was adjusted, and the anterior chamber was restored. Finally, dexamethasone-tobramycin ophthalmic ointment (Alcon) was applied to the conjunctival sac.

### Visual acuity examination

Uncorrected distance visual acuity (UDVA) (logMAR) at 5 meters, uncorrected intermediate visual acuity (UIVA) (logMAR) at 80 cm, and uncorrected near visual acuity (UNVA) (logMAR) at 40 cm were measured. Best-corrected distance visual acuity (BCDVA) (logMAR) was also assessed.

### Questionnaire survey

The first part of the questionnaire utilized the Chinese version of the Visual Function Index-11R (VF-11R) (maximum score of 50) to evaluate postoperative visual function and visual quality in daily life. Responses for each item were graded on a 5-point scale based on difficulty level, i.e., “no difficulty,” “a little difficulty,” “moderate difficulty,” “pronounced difficulty” and “impossibility to perform”, corresponding to scores of 5 to 1, respectively. The sum of scores from all items constituted the total score for this questionnaire. Visual function index 14 (VF-14) was originally developed to assess vision-specific activity limitations in patients with cataract and the outcomes of cataract surgery (4), and has been widely used to report patient outcomes. The VF-11R is a modified Chinese version of VF-14, which has been verified to be suitable for assessing the outcome of cataract surgery in the Chinese population (5). The second portion of the satisfaction questionnaire comprised 10 items (maximum score of 50) designed to assess postoperative satisfaction, quality of vision, spectacle independence and the incidence rates of glare and halos from light. The responses were scored from 5 to 1, based on satisfaction level: “very satisfied”, “satisfied”, “slightly dissatisfied”, “dissatisfied” and “very dissatisfied” (or “never”, “rarely”, “sometimes”, “often” and “always” satisfied). The total score including all questions was determined as the final score in this survey.

### Statistical methods

Statistical analysis was performed using SPSS version 22.0 or higher. Measurement data were described using mean, standard deviation, median, minimum, maximum, and interquartile range. Categorical/ordinal data were described using frequency (percentage) or number. All statistical tests (unless otherwise specified) were two-sided. A *p*-value of less than 0.05 was considered statistically significant for differences between the experimental and control groups. Conversely, a *p*-value greater than or equal to 0.05 indicated no statistically significant difference. The normality of measurement data was first assessed by the Shapiro-Wilk test. For data conforming to a normal distribution, intergroup comparisons were performed by the independent samples *t*-test. For data not conforming to a normal distribution, the Mann-Whitney U test was used. Differences in measurement data across multiple time points within or between groups were analyzed by repeated measures analysis of variance (ANOVA) or the Generalized Estimating Equations (GEE) model. Intergroup comparisons of categorical/ordinal data were generally performed by the Chi-square test or Fisher’s exact test.

## Results

### Baseline patient characteristics

Record the pupil diameters of patients under photopic and scotopic conditions, all patients had normal pupil function. The mean photopic pupil diameters in the AM3UH group and 839MP group were (3.12 ± 0.98) mm and (3.17 ± 0.82) mm, respectively, with no significant difference between the two groups. There were no statistically significant differences between the two groups in terms of axial length, age, and gender preoperatively (*P* > 0.05), indicating similar distributions in demographic data and establishing comparability between the groups. The detailed statistical results are shown in Table 1.

**TABLE 1:** Preoperative baseline data of cataract patients in the two groups.

Groups	Eyes	Pupil diameter (mm, Mean ± SD)	Axial length (mm, Mean ± SD)	Age (years, Mean ± SD)	Gender (cases, male/female)
AM3UH group	69	3.12 ± 0.98	24.24 ± 1.61	66.78 ± 8.16	34/35
839MP group	62	3.17 ± 0.82	25.01 ± 2.47	64.02 ± 10.02	27/35
*Z*-value		−0.772	1.75	−1.41	χ^2^ = 0.43
*P*-value		0.440	0.08	0.16	0.51

### Visual acuity

There was no statistically significant difference in preoperative UDVA between the two groups (*P* > 0.05). Within each group, UDVA at 3 months postoperatively showed a statistically significant improvement compared to preoperative levels (*P* < 0.05). There were no statistically significant differences in UDVA, UNVA and BCDVA between the two groups at 3 months after surgery (*P* > 0.05). UIVA was significantly better in the 839MP group than in the AM3UH group (*P* < 0.05) (Table 2).

**TABLE 2:** Comparison of preoperative and postoperative visual acuity of cataract patients in the two groups (log MAR, $\bar{x}$ ± s).

Groups	Eyes	Preop.	UDVA of 3 months postop.	UIVA of 3 months postop.	UNVA of 3 months postop.	CDVA of 3 months postop.
AM3UH group	69	0.93 ± 0.38	0.14 ± 0.19	0.13 ± 0.12	0.15 ± 0.1	0.09 ± 0.11
839MP group	62	0.90 ± 0.47	0.12 ± 0.08	0.07 ± 0.08	0.12 ± 0.06	0.05 ± 0.04
*Z*-value		−0.70	0.20	−2.35	−1.39	−0.89
*P*-value		0.54	0.84	0.02	0.16	0.40

### Questionnaire survey

**FIGURE 1:**
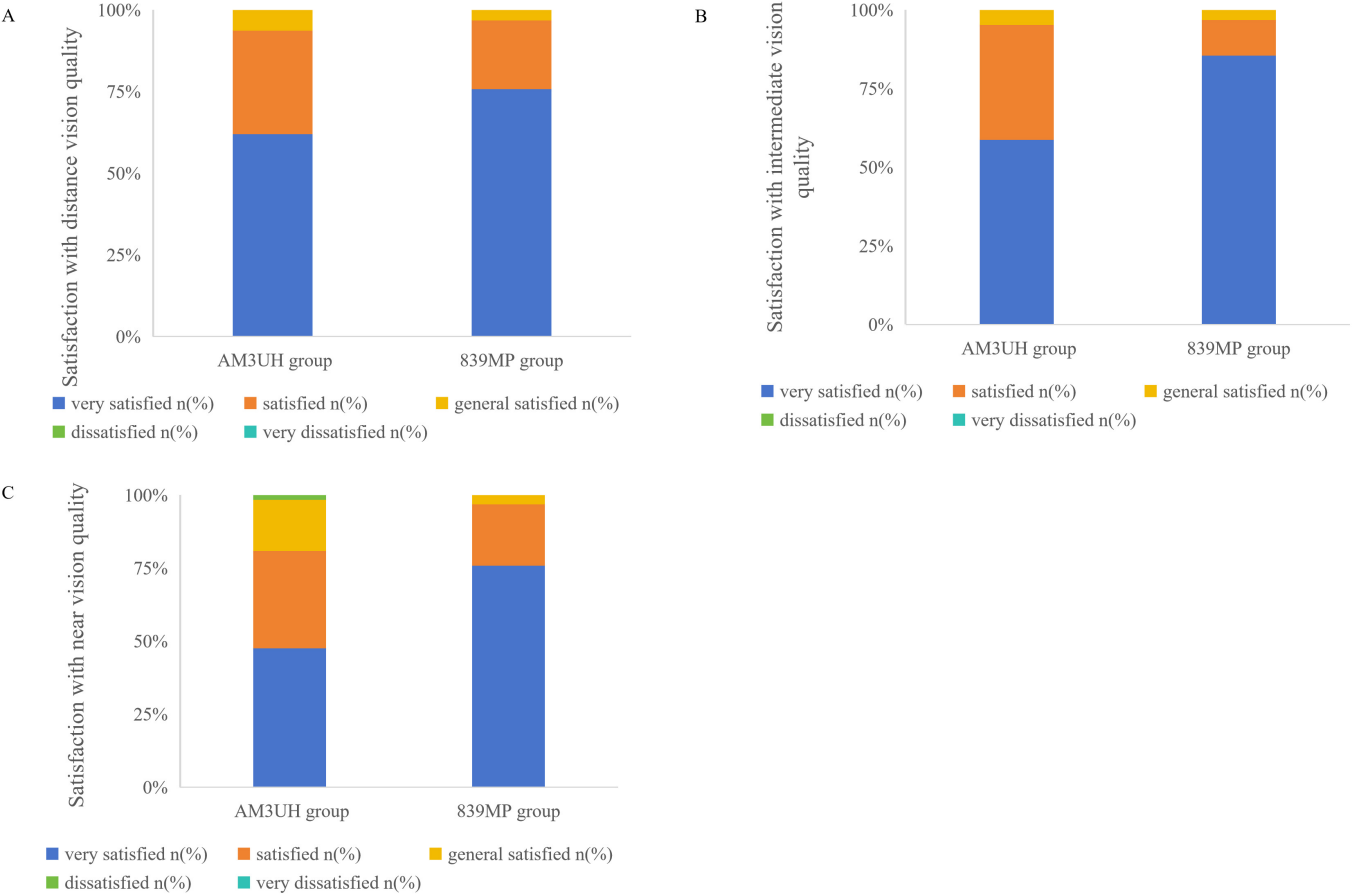
Comparison of distance, intermediate and near visual acuity satisfaction between diffractive multifocal intraocular lens AM3UH group and 839MP group at 3 months after surgery **(A)**, Satisfaction with distance visual quality; **(B)** Satisfaction with intermediate visual quality; **(C)** Satisfaction with near visual quality.

Three months after surgery, the scores obtained with the VF-11R questionnaire in the AM3UH group were 49.23 ± 1.22, versus 48.87 ± 1.61 in the 839MP group, with no statistically significant difference between the two groups (*Z* = −1.522, *P* = 0.128 > 0.05). Regarding patient satisfaction with distance visual symptoms in the surgical eyes at 3 months postoperatively, the distributions of satisfaction levels (very satisfied, satisfied, neutral, dissatisfied and very dissatisfied) for distance visual quality were 39, 20, 4, 0, and 0 cases in the AM3UH group, respectively, and 47, 13, 2, 0, and 0 cases in the 839MP group, respectively. The intergroup difference was not statistically significant (*Z* = 2.32, *P* = 0.357 > 0.05). For satisfaction with intermediate visual quality, the distributions were 37, 23, 3, 0, and 0 cases in the AM3UH group, respectively, and 53, 7, 2, 0, and 0 cases in the 839MP group, respectively, showing a statistically significant intergroup difference (*Z* = 11.77, *P* = 0.002 < 0.05). For satisfaction with near visual quality, the distributions were 30, 21, 11, 1, and 0 cases in the AM3UH group, respectively, and 47, 13, 2, 0, and 0 cases in the 839MP group, respectively, also demonstrating a statistically significant intergroup difference (*Z* = 12.82, *P* = 0.002 < 0.05). Satisfaction rates were higher in the 839MP group than in the AM3UH group for both intermediate and near visual quality (Figure 1).

**FIGURE 2:**
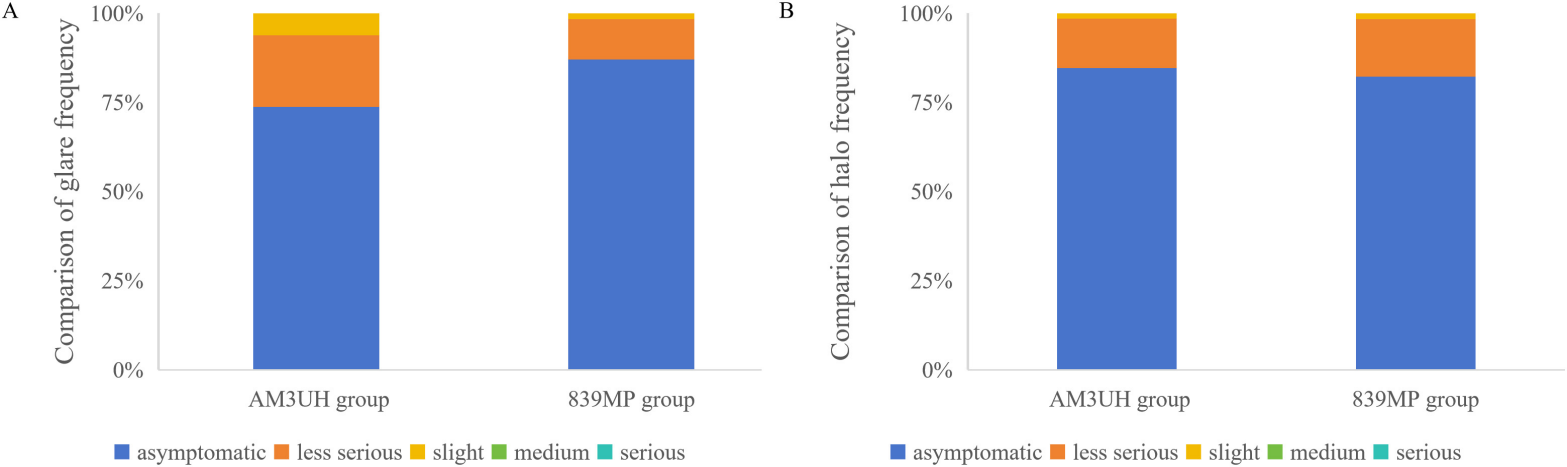
Comparison of visual interference symptoms between the diffractive multifocal IOL AM3UH group and the 839MP group at 3 months after surgery **(A)**, Comparison of glare frequency; **(B)** Comparison of halo frequency.

There were no statistically significant differences between the two groups in terms of visual symptoms such as glare and halos in the surgical eyes at 3 months postoperatively (*P* > 0.05). Specifically, for glare disturbance, the distribution of cases across severity grades was 48, 13, 4, 0, and 0 in the AM3UH group, versus 54, 7, 1, 0, and 0 in the 839MP group, with no statistically significant difference between the groups (*Z* = 3.70, *P* = 0.06 > 0.05). Similarly, for halo disturbance, the distributions were 55, 9, 1, 0, and 0 in the AM3UH group, respectively, and 51, 10, 1, 0, and 0 in the 839MP group, respectively, also showing no statistically significant difference between the groups (*Z* = 0.40, *P* = 0.84 > 0.05). Most patients with glare or halos were either asymptomatic or had mild symptoms. The number of patients experiencing glare was lower in the 839MP group than in the AM3UH group, while slightly more patients reported halos in the 839MP group compared with the AM3UH group (Figure 2).

**FIGURE 3:**
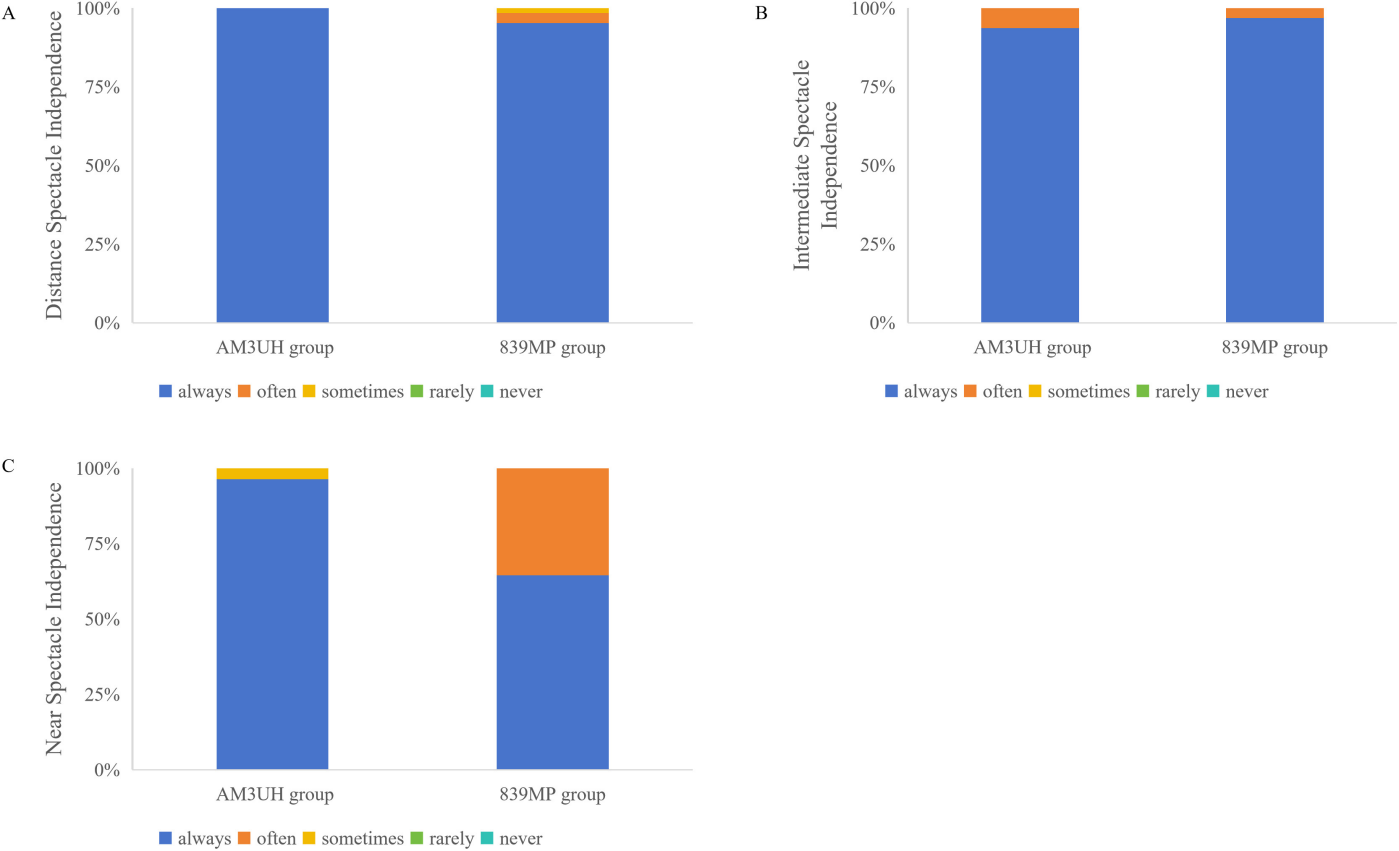
Comparison of spectacle independence rate between diffractive multifocal IOL AM3UH group and 839MP group at 3 months after surgery **(A)**, Distance Spectacle independence; **(B)** Intermediate Spectacle independence; **(C)** Near Spectacle independence.

There were no statistically significant differences in spectacle independence rates for distance, intermediate, and near vision between the two groups at 3 months postoperatively (*P* > 0.05). Specifically, for far vision, the distributions of spectacle independence frequency (always, often, sometimes, rarely, never) were 69, 0, 0, 0, and 0 cases in the AM3UH group, respectively, and 59, 2, 1, 0, and 0 cases in the 839MP group, respectively, with no statistically significant difference between the groups (*Z* = 2.97, *P* = 0.103 > 0.05). For intermediate vision, the distributions were 65, 4, 0, 0, and 0 cases in the AM3UH group, respectively, and 60, 2, 0, 0, and 0 cases in the 839MP group, respectively, showing no statistically significant difference between the groups (*Z* = 0.49, *P* = 0.683). Similarly, for near vision, the distributions were 58, 9, 2, 0, and 0 cases in the AM3UH group, respectively, and 40, 22, 0, 0, and 0 cases in the 839MP group, respectively, with no statistically significant difference between groups (*Z* = 3.27, *P* = 0.180 > 0.05) (Figure 3).

## Discussion

Cataract is the leading cause of blindness worldwide. The only effective treatment currently available is surgical removal of the opacified natural lens and implantation of an artificial IOL. Advances in surgical techniques and innovations in IOL design have brought hope for improving postoperative visual quality in cataract patients. Full-range refractive surgery aimed at achieving spectacle independence has gradually become a trend in cataract treatment. Driven by the increasing demand for high-quality postoperative vision among cataract patients, MIOLs have thus emerged. However, use of these functional IOLs is inevitably associated with visual disturbances, including glare and halos. These issues are typically caused by an incomplete or compromised optical system, such as the presence of cataract and the implantation of an MIOL (6).

Given the limitations associated with different optical principles, IOLs incorporating hybrid designs have emerged. These lenses aim to provide spectacle independence while minimizing adverse optical phenomena. Approved for marketing in 2022, AM3UH is a MIOL that combines a hybrid refractive-diffractive design with EDOF. Its anterior surface utilizes an EDOF aspheric technology to achieve continuous focal extension, while its posterior surface features a bifocal diffractive ring design to provide distinct distance and near focal points. By integrating the bifocal technology with an EDOF design, this lens aims to deliver full-range continuous vision.

The AT LISA tri 839MP trifocal IOL, approved in 2015, is designed to provide clear vision at distance, intermediate, and near focal points, thereby reducing dependence on spectacles (7, 8). Its diffractive ring pattern is intended to minimize glare (9). Compared with monofocal IOLs, MIOLs or trifocal IOLs are more likely to cause visual disturbance symptoms in patients after implantation (10, 11). Therefore, this study compared AM3UH and AT LISA tri 839MP for their impact on postoperative subjective and objective visual quality in cataract patients. The findings aim to provide evidence to support clinical decision-making regarding the selection of these two IOL types and their associated potential risks.

This multicenter, retrospective, prospective, bidirectional cohort study included 69 eyes implanted with the EDOF hybrid refractive-diffractive bifocal IOL AM3UH and 62 eyes implanted with the hybrid refractive-diffractive trifocal IOL AT LISA tri 839MP. The results demonstrated that UDVA at 3 postoperative months showed statistically significant improvement compared with preoperative levels in both the AM3UH and 839MP groups (*P* < 0.01). No statistically significant differences were observed between the two groups in terms of postoperative UDVA, UNVA and BCDVA at 3 months after surgery. However, UIVA was better in the 839MP group than in the AM3UH group, with a statistically significant difference (*P* < 0.05). These findings suggest that both the AM3UH and 839MP IOLs can provide age-related cataract patients with good distance and near vision. Notably, the 839MP group demonstrated superior intermediate visual function over the AM3UH group, thereby enhancing postoperative quality of life in patients with intermediate vision demands. The optical design of 839MP theoretically provides better intermediate vision (12). Previous studies (13, 14) have also confirmed that the postoperative UIVA of the 839MP is superior to that of bifocal IOLs, which is consistent with our findings that the 839MP provides better intermediate visual acuity than the AM3UH. In contrast, multiple studies have shown that, compared with trifocal IOLs, EDOF IOLs may provide better image quality at intermediate and near distances (15); non-randomized studies support this and suggest that EDOF IOLs may offer better visual acuity at intermediate distances than multifocal IOLs (16). Our results, however, contradict these findings, which differs from the expectation that EDOF lenses are typically marketed as providing “optimized intermediate vision.” We refer to this as the “intermediate vision paradox.” Some studies have reported no significant difference in UIVA between trifocal and EDOF IOLs (4). Monaco et al. (5) found that trifocal IOLs were slightly superior to EDOF IOLs, whereas Webers et al. (17) reported that EDOF IOLs were slightly superior to trifocal IOLs. In light of these discrepant findings, we hypothesize that: the AT LISA tri 839MP incorporates an additional +1.66 D add power for intermediate distance, whereas the AM3UH is a hybrid refractive-diffractive EDOF IOL with only a near add of +2.8 D, and its depth-of-focus extension design may focus more on a continuous far-to-intermediate range rather than peak visual acuity at a specific intermediate point. Furthermore, differences in the definition of intermediate distance and testing conditions across studies may influence outcomes. For example, Karuppiah et al. (4) measured intermediate visual acuity at 60 cm and found that the EDOF IOL group was significantly superior to the trifocal IOL group; in contrast, we measured intermediate visual acuity at 80 cm, which may not be the optimal distance for the AM3UH IOL.

The VF-11R is a validated, reliable, and effective instrument for measuring functional impairment caused by cataracts (18). In this study, no statistically significant difference was observed in VF-11R scores between the two patient groups, indicating that both groups achieved good postoperative visual quality.

Furthermore, one study found that the visual function score was better in the trifocal group (5). In our study, postoperative subjective questionnaire surveys revealed that both the AM3UH and 839MP groups reported high levels of satisfaction with distance, intermediate, and near visual quality, which is consistent with the findings of Tavassoli et al. (19). No statistically significant difference was observed between the two groups in terms of distance visual quality satisfaction, whereas the 839MP group showed superior satisfaction for both intermediate and near visual quality compared to the AM3UH group. Evidence indicates that participants receiving trifocal IOLs are less dependent on glasses for near tasks than those receiving EDOF IOLs. There was no statistically significant difference between the two groups in the frequency of visual disturbances. This is in line with Tavassoli et al. (19), who reported that although adverse optical phenomena were observed in both groups, the difference between the groups was not statistically significant. Postoperatively, only a few patients in either group experienced glare or halos; the low incidence of visual disturbances may be attributed to the aspheric, step-progressive diffractive design between the diffractive rings on the optic (20). Some studies have found that the percentages of glare and halos are higher with trifocal IOLs than with EDOF IOLs (4). In our results, however, the AM3UH group had slightly more patients with glare than the 839MP group, which may be related to the AM3UH’s design featuring a refractive anterior surface and a diffractive ring posterior surface. However, the borderline *P-*value for glare (0.06) should be interpreted with caution. The lack of blinding in patient-reported outcomes may have influenced subjective symptom ratings. Moreover, with a larger sample size, this difference could reach statistical significance. Future studies with adequate power and blinded assessment are warranted. The 839MP group had slightly more patients with halos than the AM3UH group, likely due to its diffractive design, which is consistent with previous reports. It has been reported that the brain requires at least 3 months to adapt to multifocal IOLs (21, 22), and subjective optical disturbances can largely be resolved through neuroadaptation; within 6–12 months, most patients experience symptom improvement, and typically no specific intervention is required (23). Therefore, a longer observation period is needed for both groups. There was no statistically significant difference between the two groups in spectacle independence rates for distance, intermediate, and near vision, which is consistent with the findings of Bian et al. (14).

The limitations of this study include a small sample size, a short follow-up period, and limited postoperative subjective and objective visual quality assessment metrics, such as the absence of comparative variables including contrast sensitivity, defocus curve, and the tolerance of the intraocular lens to minimal refractive outcomes (myopic or hyperopic). Furthermore, this was a non-randomized study, and both patients and surgeons were aware of the lens type, which may introduce bias. In future work, we will improve the study design by conducting large-scale, multicenter studies with larger sample sizes and a blinded design to more reliably evaluate the optical phenomena associated with different IOL types. Extended follow-up periods and additional outcome measures will be implemented to obtain more robust data. Despite these limitations, our study provides clinical data comparing the AT LISA tri 839MP and AM3UH IOLs, which may serve as a reference for selecting IOLs for patients with different visual needs. In conclusion, both AM3UH and 839MP can provide good visual acuity and spectacle independence in patients with age-related cataract after surgery. 839MP resulted in superior intermediate visual acuity compared with AM3UH, along with higher patient satisfaction regarding intermediate and near vision. This advantage is likely attributable to the +1.66 D addition for intermediate vision in the 839MP design, which provides enhanced intermediate visual performance, and the +3.33 D addition for near vision, which is higher than the +2.8 D addition of AM3UH, contributing to better near visual acuity. No statistically significant differences were observed between the two groups in terms of VF-11R scores or spectacle independence rates, indicating that the IOL AM3UH provided slightly worse intermediate and near vision than 839MP, although it did not affect postoperative intermediate and near vision and glass wearing. Personalized lens selection is an important part of clinical work. This study aimed to provide additional insights for clinical workers.

## Data Availability

The raw data supporting the conclusions of this article will be made available by the authors, without undue reservation.
